# Fatal Tuberculosis in a Free-Ranging African Elephant and One Health Implications of Human Pathogens in Wildlife

**DOI:** 10.3389/fvets.2019.00018

**Published:** 2019-02-06

**Authors:** Michele A. Miller, Peter Buss, Eduard O. Roos, Guy Hausler, Anzaan Dippenaar, Emily Mitchell, Louis van Schalkwyk, Suelee Robbe-Austerman, W. Ray Waters, Alina Sikar-Gang, Konstantin P. Lyashchenko, Sven D. C. Parsons, Robin Warren, Paul van Helden

**Affiliations:** ^1^Division of Molecular Biology and Human Genetics, Faculty of Medicine and Health Sciences, DST-NRF Centre of Excellence for Biomedical Tuberculosis Research, South African Medical Research Council Centre for TB Research, Stellenbosch University, Cape Town, South Africa; ^2^Veterinary Wildlife Services, South African National Parks, Kruger National Park, Skukuza, South Africa; ^3^Department of Research and Scientific Services, National Zoological Gardens, South African Biodiversity Institute, Pretoria, South Africa; ^4^Faculty of Veterinary Science, University of Pretoria, Onderstepoort, South Africa; ^5^Department of Agriculture, Forestry and Fisheries, Skukuza State Veterinary Office, Skukuza, South Africa; ^6^National Veterinary Services Laboratories, Animal Plant Health Inspection Service, United States Department of Agriculture, Ames, IA, United States; ^7^National Animal Disease Center, Agricultural Research Service, United States Department of Agriculture, Ames, IA, United States; ^8^Chembio Diagnostic Systems, Inc. Medford, NY, United States

**Keywords:** African elephant, anthroponosis, *Loxodonta africana*, *Mycobacterium tuberculosis*, one health, tuberculosis, wildlife disease

## Abstract

Tuberculosis (TB) in humans is a global public health concern and the discovery of animal cases of *Mycobacterium tuberculosis* (Mtb) infection and disease, especially in multi-host settings, also has significant implications for public health, veterinary disease control, and conservation endeavors. This paper describes a fatal case of Mtb disease in a free-ranging African elephant (*Loxodonta africana*) in a high human TB burden region. Necropsy revealed extensive granulomatous pneumonia, from which Mtb was isolated and identified as a member of LAM3/F11 lineage; a common lineage found in humans in South Africa. These findings are contextualized within a framework of emerging Mtb disease in wildlife globally and highlights the importance of the One Health paradigm in addressing this anthroponotic threat to wildlife and the zoonotic implications.

## Introduction

Tuberculosis (TB) is the leading cause of death from a bacterial infectious disease in humans. Caused by *Mycobacterium tuberculosis* (Mtb), approximately one third of the global population is thought to be infected, with high burdens in lower income countries, including much of Africa and Asia (1). The socioeconomic, and public health costs can be staggering; for example, in South Africa, where 244,053 people were reported with TB in 2016, the national TB program budget (US$244 million) was a significant proportion of the national budget (2). However, these figures neglect the potential impact of human TB on other incidental host species, especially livestock, and wildlife.

Many high human TB burden countries are dependent on animal-related industries such as agriculture and tourism to support their economy. With encroachment of human settlements into land previously used for agriculture or natural habitats, there are increased opportunities for disease transmission at the interface between animals and people. Despite the emerging field of “One Health” and growing knowledge of the zoonotic risks of animal diseases (3), few studies have assessed the impact of human diseases on animals (4). Tuberculosis, caused by Mtb, has been reported in cattle in rural areas of Africa, including the Eastern Cape Province of South Africa, as well as in captive wildlife and pets (5–9). However, infections with Mtb have been discovered only recently in free-ranging wildlife in Asia (10–12). Although animals are typically considered dead-end hosts for Mtb, there is evidence that infected elephants are capable of spreading infection to other elephants and different species, including humans (13–16). Therefore, discovery of animal cases of Mtb infection and disease, especially in free-range, multi-host settings, could have significant implications for species management, public health and veterinary disease control, and conservation endeavors. This case of Mtb disease in a free-ranging African elephant highlights the importance of applying the One Health paradigm to address anthroponoses where important human pathogens, such as Mtb, can be introduced into wildlife populations (4).

## Materials and Methods

### Case

In October 2016, the fresh carcass of an African elephant bull (estimated age 45 years) was found near the tourist and staff camp of Tshokwane (S24° 47′ 9.24″ E 31° 51′ 33.12″), in the Kruger National Park (KNP), South Africa. The animal was in poor body condition with no external wounds or injuries. Another bull elephant was observed in close proximity to this animal but appeared to be in good body condition. Samples taken at necropsy included sections of lungs and lymph nodes that were frozen for mycobacterial culture and placed in 10% buffered formalin for histopathology, impression smears of lesions for acid-fast stain cytology, and heart blood for serological tests. Safety precautions and biosecurity measures were implemented during the necropsy. Infected lungs and other organs were removed from the carcass and incinerated.

### Serological Assays

Whole blood was collected from the heart into serum separator tubes, which formed a clot, and then serum was harvested by centrifuged at 3,000 x g for 10 min. Serology to detect the presence of antibodies to Mtb complex (MTBC)-specific antigens was performed using the Chembio DPP VetTB assay (Chembio Diagnostic Systems, Inc., Medford, NY) and the multi-antigen print immunoassay (MAPIA) (17, 18).

### Mycobacterial Culture, Speciation, and Whole Genome Sequencing

Lung and lymph node tissues were processed for mycobacterial culture using the BACTEC™ Mycobacteria Growth Indicator Tube (MGIT™) system in a BSL3 laboratory (19). An aliquot from each of the MGIT, containing acid-fast positive bacteria, was genetically speciated by PCR (20). The isolate was re-cultured and used for DNA extraction as previously described (21). Whole genome sequencing was performed using the NexteraXT library preparation kit (Illumina, San Diego, CA, USA) and sequenced using 2x250 paired end chemistry on a MiSeq (Illumina). Whole genome sequences are available under BioProject ID: PRJNA430907. See Supplementary Methods for additional details on whole genome sequencing data analysis.

## Results

**Figure 1:**
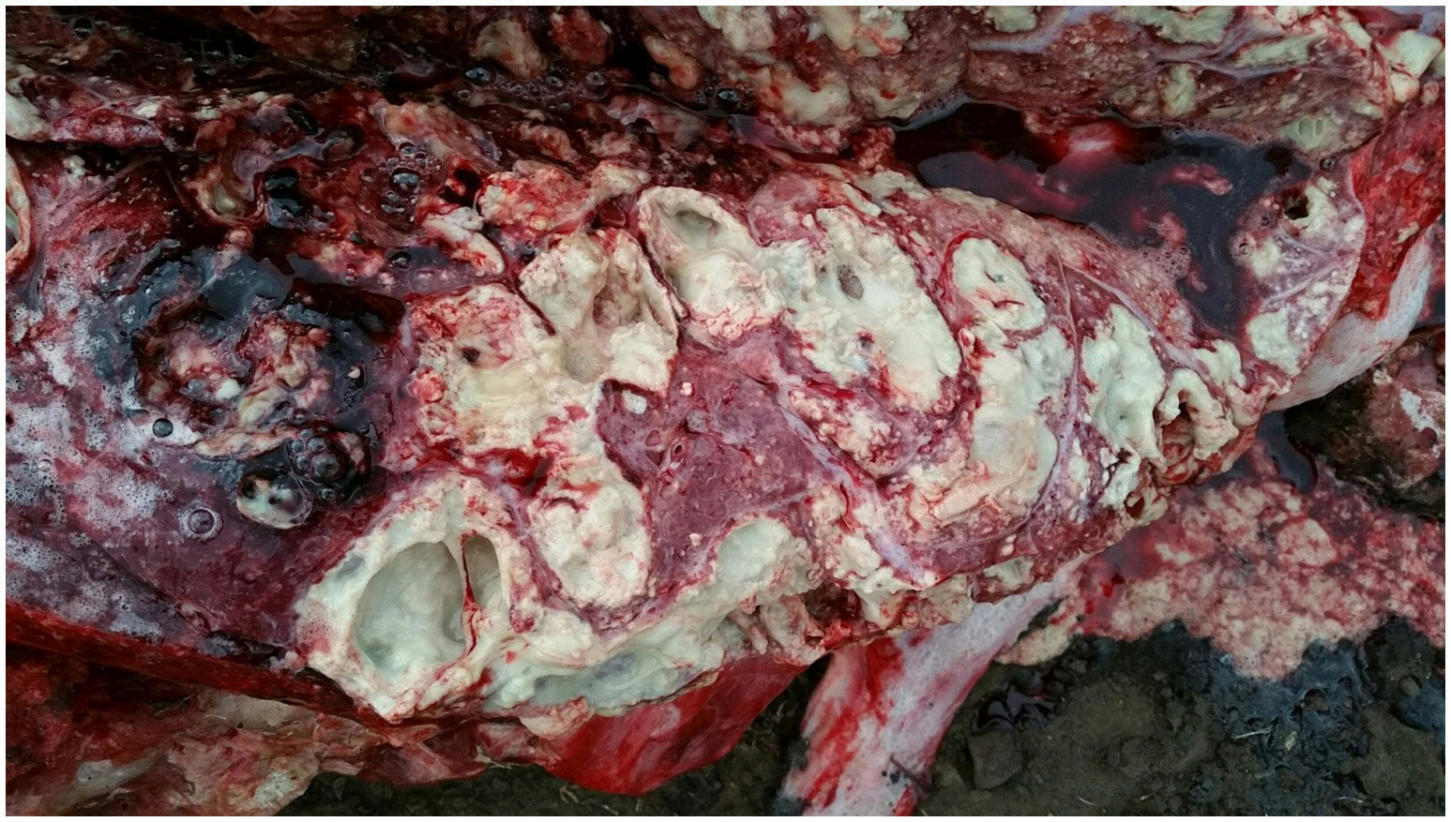
Image of gross pathological changes in lungs of African elephant with Mtb disease. Lung lesions consisted of multifocal to coalescing encapsulated cavities (10–15 cm in diameter).

**Figure 2:**
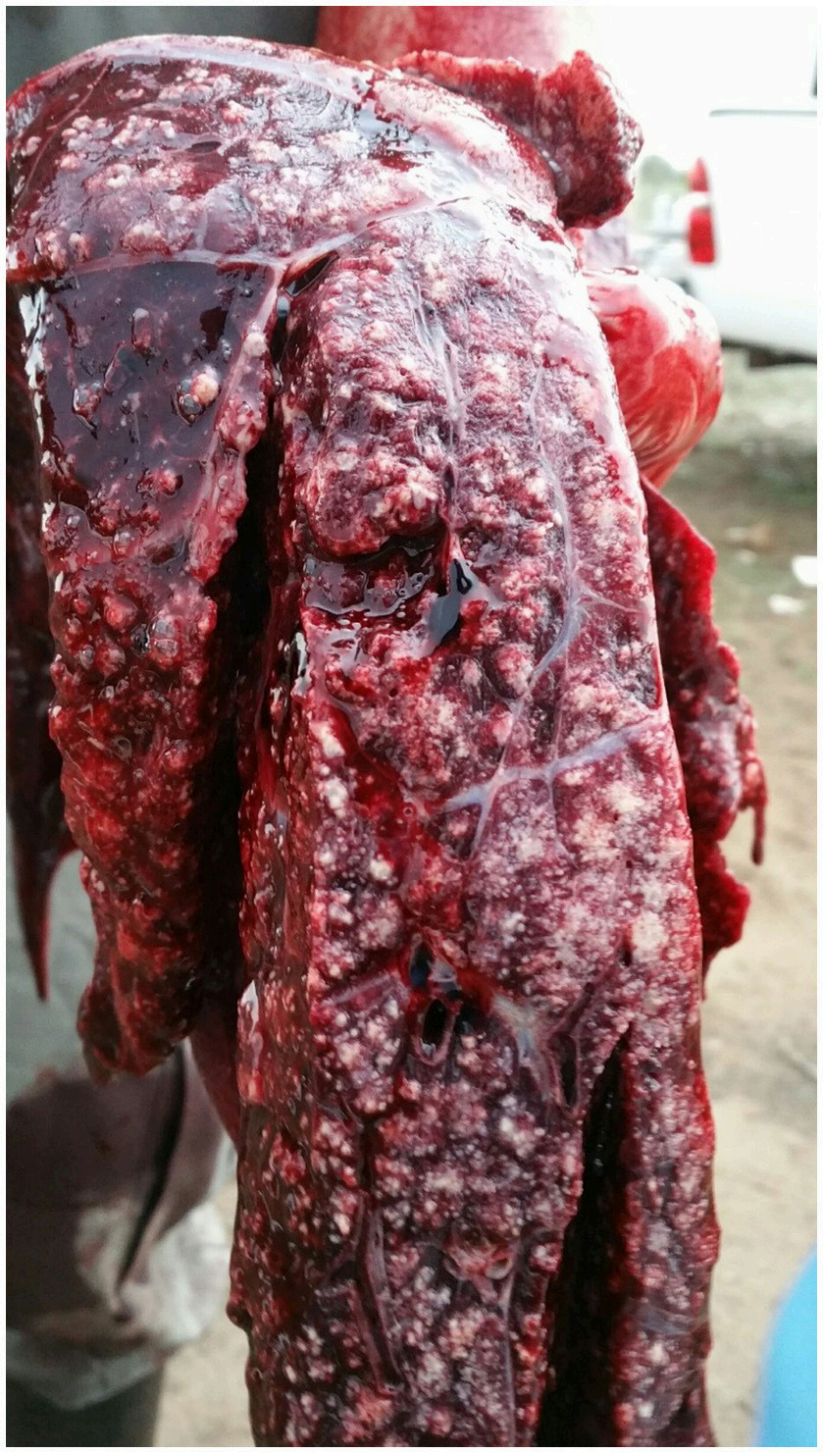
Image of gross pathological changes in lungs of African elephant with Mtb disease. Lung lesions comprised a mixture of cavitating lesions and military focal granulomas.

**Figure 3:**
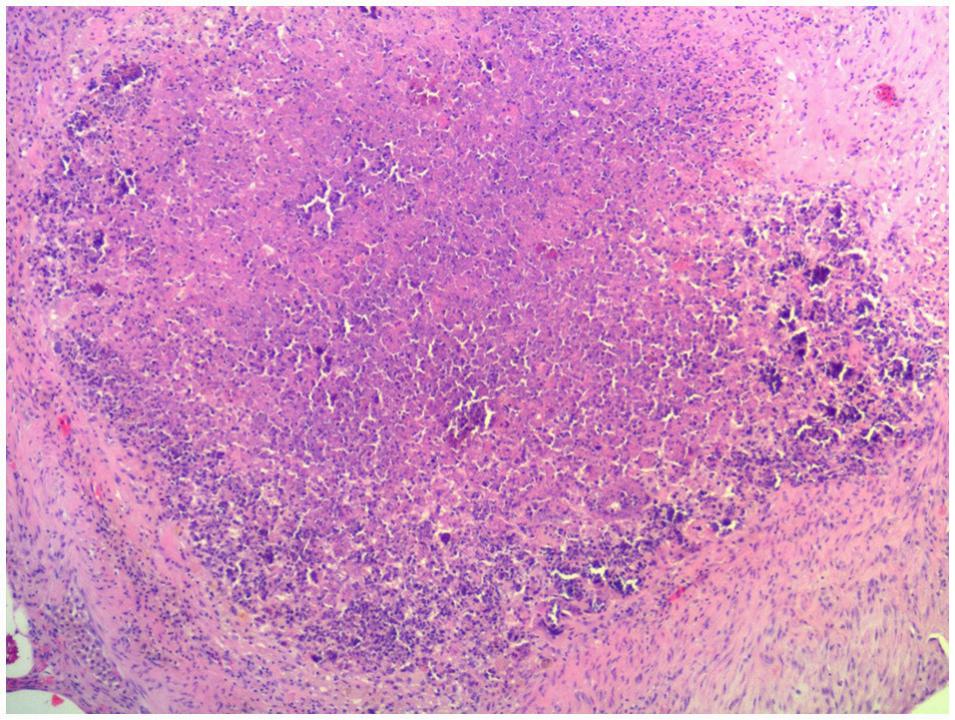
Image of representative histopathological changes in lungs of African elephant with Mtb disease. Granulomas comprised central foci of variably mineralized necrotic debris and clusters of acid-fast positive bacilli encapsulated in variably thick layers of macrophages and epithelioid cells, mixed with small numbers of multinucleate giant cells, lymphocytes, and plasma cells.

At necropsy, an estimated 80% of the left lung and 40–50% of the right lung consisted of multifocal to coalescing encapsulated cavities (10–15 cm in diameter) (Figure 1). The lungs contained a mixture of cavitating lesions and miliary focal granulomas (Figure 2). Impression smears showed clusters of acid-fast positive bacilli. The primary histological finding was multifocal pyogranulomatous pneumonia. Granulomas comprised central foci of variably mineralized necrotic debris and clusters of acid-fast positive bacilli encapsulated in variably thick layers of macrophages and epithelioid cells (many of which contained haematoidin pigment), mixed with small numbers of multinucleate giant cells, lymphocytes, and plasma cells (Figure 3). Similar lesions were found in the bronchial lymph nodes.

**Figure 4:**
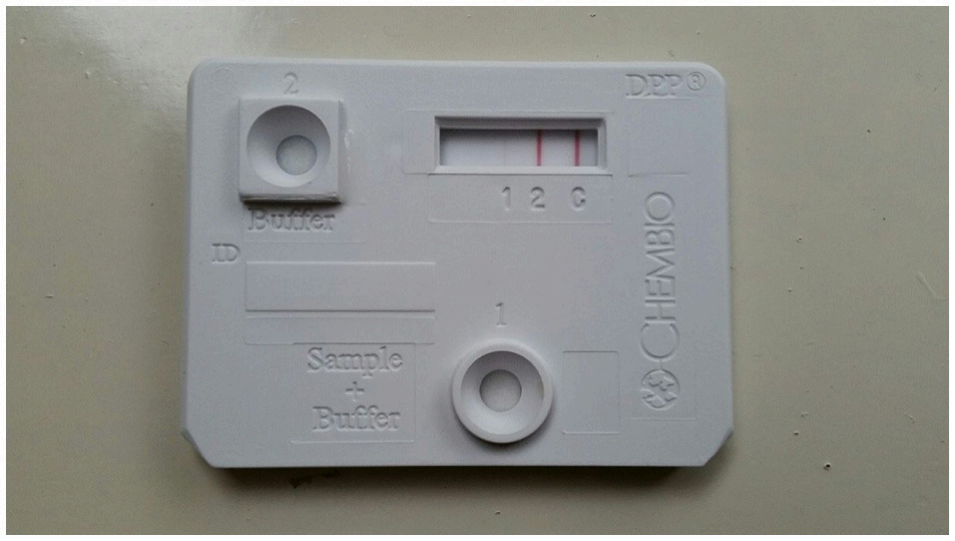
Image of DPP VetTB assay using serum from African elephant with Mtb disease. Serology methods were performed as previously described (18). Visible band at test line 2 demonstrates presence of antibodies to ESAT-6/CFP10 fusion protein, which are Mtb complex specific antigens.

**Figure 5:**
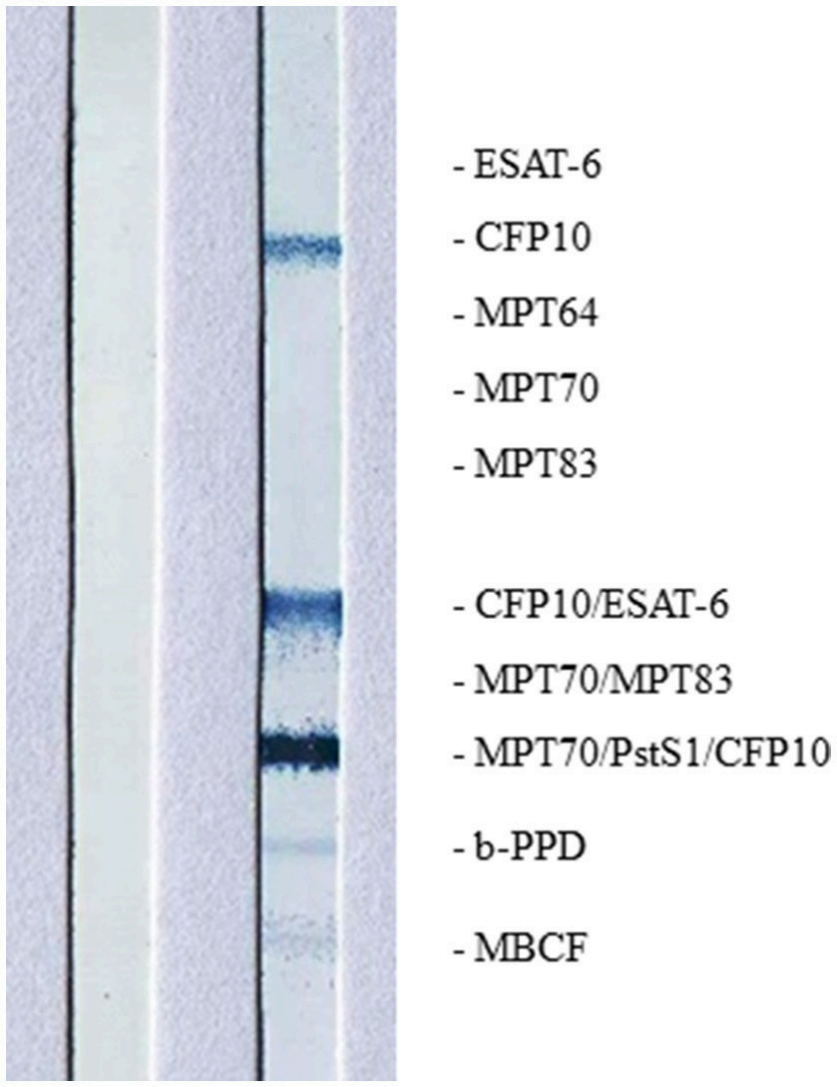
Image of MAPIA results are shown for African elephant diagnosed with Mtb disease (right strip) and a negative control African elephant (left strip); names and positions of immobilized antigens are shown on the right margin; visible bands on the strip indicate the presence of IgG antibody to corresponding antigens.

High intensity of the test line in the Chembio DPP VetTB assay suggested the presence of IgG antibodies to MTBC-specific fusion antigen ESAT-6/CFP10 (Figure 4). In addition, the presence of high intensity lines for the MTBC-specific antigens including CFP10 protein, ESAT-6/CFP10 fusion protein, and DID65 fusion (MPT70/PstS1/CFP10) protein (17), using the multi-antigen print immunoassay (MAPIA), supported a presumptive diagnosis of TB (Figure 5) (18, 22).

**Figure 6:**
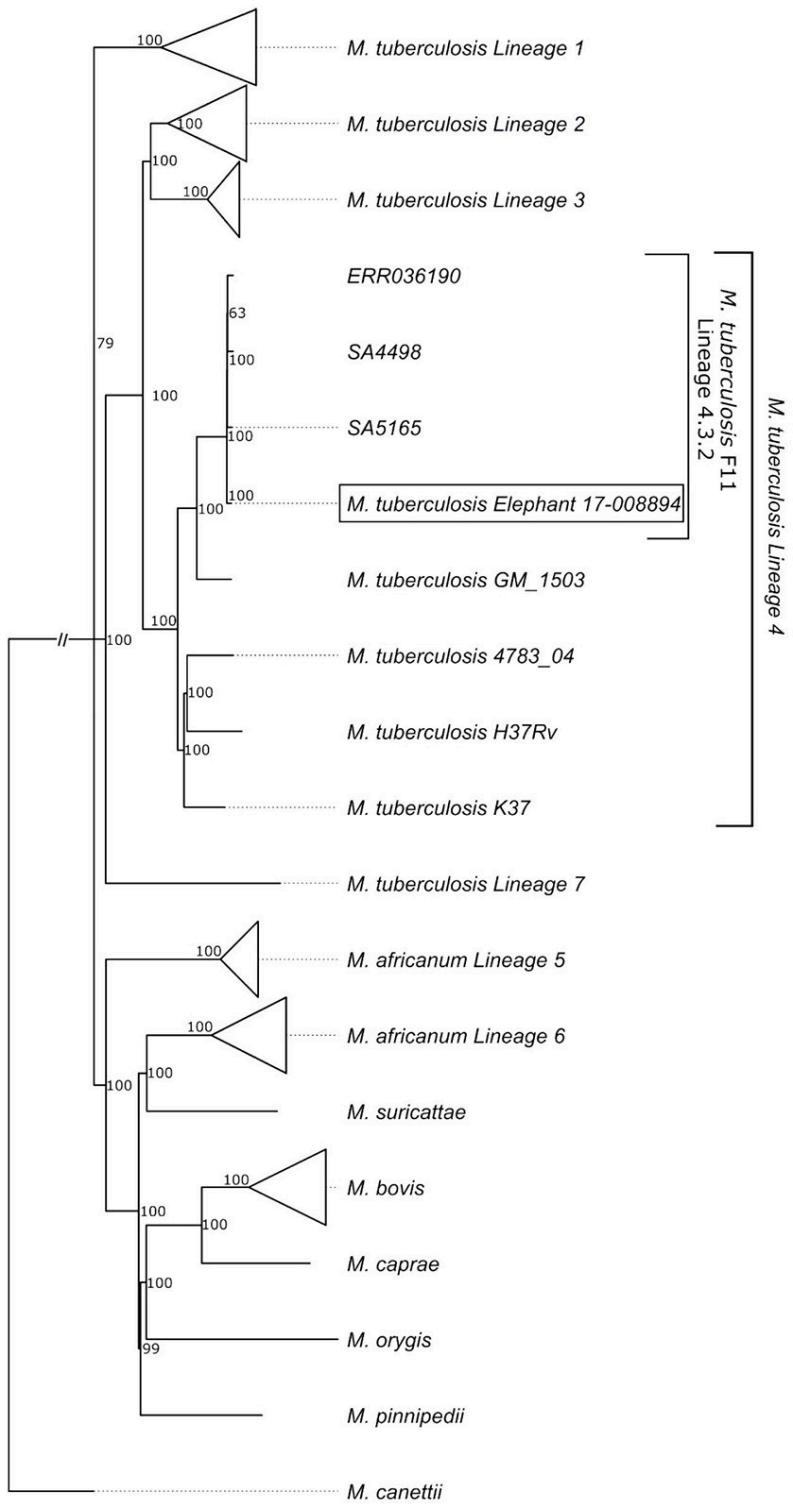
Phylogenetic tree showing the relationship of the elephant Mtb isolate to other known human Mtb isolates; with clustering of the elephant isolate in the LAM3/F11 family.

*Mycobacterium tuberculosis* was isolated from lung, pooled head (retropharyngeal, mandibular), thoracic (mediastinal, tracheobronchial), and mesenteric lymph nodes, which were all the samples collected for mycobacterial culture due to the presence of gross lesions. Spoligotyping to confirm infection (rather than laboratory contaminant) and characterization of the separate culture isolates was performed. The isolates were characterized as belonging to the SIT33/LAM3/F11 family (23, 24) (octal code 776177607760771). Compared to other Mtb isolates previously reported from elephants, the whole genome sequence was unique (BioProject ID is PRJNA430907; BioSample accession is SAMN08380889; http://www.ncbi.nlm.nih.gov/biosample/8380889). However, it clustered in the LAM3/F11 family commonly found in human TB patients in South Africa (24) (Figure 6; Table S1).

## Discussion

This is the first confirmed case of Mtb disease in a free-ranging African elephant and suggests that this anthroponosis may be a greater threat to wildlife populations in Africa than previously recognized. Previously, a presumptive diagnosis of TB was made in a free-ranging African elephant with past human contact in Kenya, but culture of affected tissues was not performed and infection and identity of the suspected MTBC organism were not confirmed (25). Cases of Mtb disease have been reported in free-ranging Asian elephants in Sri Lanka and India, supporting the emergence of this important anthroponotic disease (11, 12, 26).

*Mycobacterium bovis* is endemic in KNP, where multiple host species are infected (27, 28). In the case described in this report, *M. bovis* was initially suspected as the most likely causative agent, although *M. bovis* had not previously been reported in elephants in the park or elsewhere in southern Africa. Investigation of the source of the Mtb in our case was confounded by the likelihood that the elephant may have been infected for many years prior to death, based on the extensive pulmonary pathology. Chronic Mtb infection has been documented for at least 9 years in captive elephants (18, 29). Despite these constraints, a Mtb surveillance program for elephants has been implemented in KNP, which includes retrospective serological testing using DPP VetTB as well as prospective bronchoalveolar lavage for mycobacterial culture.

Since the Mtb isolate from the African elephant clustered with the F11 strain commonly found in human TB patients in South Africa (24), we hypothesize that indirect contact may have occurred through human-derived contaminated food or infectious biological discharge. The carcass was found close to a popular rest stop and staff camp which are frequently visited by local elephants. However, since elephants can move over vast distances and show very little respect for man-made boundaries, there could be other potential sources of infection.

This case is similar to those found in Asia with no identifiable source of Mtb found in free-ranging Asian elephants from parks where tourists have no direct contact with animals (11, 12, 26). However, it is possible that these elephants may have had access to human waste in the park or human settlements along the boundaries. Alternatively, although less likely, Mtb may have been transmitted from another infected animal (29–31).

Transmission of Mtb between humans and from humans to animals has been linked to prolonged close contact, primarily through aerosols (32, 33). A report from Spain described three separate cases of Mtb, between 2007 and 2009, in cattle herds that were linked to active tuberculosis in farm staff (34). Similarly, Mtb has been isolated from cattle and domestic pigs in a number of European countries, as well as an African elephant, agouti and tapir in a Polish zoo (35). In North American zoos, the prevalence of TB in captive Asian elephants is higher than in African elephants, which may reflect a closer association of humans with this species, especially in range countries (36). Studies of TB in captive elephants in India have shown the highest seroprevalence in temple elephants that have high human contact (37). However, recent reports describing Mtb transmission between captive wildlife species over long distances challenge this paradigm. Diagnosis of human TB in a chimpanzee, black rhinoceros, Rocky mountain goats, lar gibbon, giraffe, and tapir has been associated with Mtb*-*infected Asian elephants in the same zoos but with no documented contact (29, 38, 39).

Indirect contact through environmental contamination is recognized as a risk factor for inter-species transmission of *M. bovis*, another member of the MTBC. Cattle have been infected by sharing grazing and water sources contaminated by secretions from *M. bovis*-infected badgers, fallow and red deer, white-tailed deer, and wild boar (39–42). Transmission of *M. bovis* has occurred in cattle that were exposed to uneaten feed from infected deer after 140 days (43).

Since elephants frequently use their trunk to investigate their environment, it is possible that the elephant in the present case became infected through aerosolization of bacteria on contaminated food or domestic waste from a Mtb-infected human. Urine from infected humans may also contaminate the environment and be a source of Mtb. Studies have shown that pathogenic mycobacteria in fresh human urine could survive up to 2 weeks at 30°C and up to 6 weeks at 15°C (44). Inadequate treatment of waste water may also be a potential source, as Mtb was found in stream water in Slovakia (45). Some of the rivers in KNP also serve as water sources for local communities upstream of KNP, and therefore could be a potential source of Mtb through contamination by human waste.

Although the exact location, number and duration of potential exposures are unknown for this elephant, it is possible that it was a single and brief event. Although frequent and prolonged exposure to a person with TB disease increases the risk of TB infection in humans, infection after casual contact can occur as demonstrated by a human case after a visit to a work site three times, for <15 min per visit (46, 47). Similarly, three workers with no other TB risk factors were infected with Mtb after occupational exposure to contaminated medical waste at a treatment facility (48). The possibility of infection following a single time exposure or limited indirect contact, especially in free-ranging animals, has significant implications for our perspectives on zoonotic and anthroponotic TB transmission.

The knowledge gaps evident in this case regarding a human pathogen in a wildlife species highlights the One Health focus required to address the issue of TB at the human-animal interface. Wildlife TB is recognized as a serious barrier to animal conservation efforts in South-East Asia (49). This threat to human, captive and free-ranging wildlife health is a growing concern globally (50), with the significance of this problem shown by the Government of Nepal's endorsement of The Nepal Elephant Tuberculosis Control and Management Action Plan (2011–2015) (51). Emerging diseases with potential to affect multiple species are of particular concern since wildlife may become reservoirs that impact domestic animal and human health, and biodiversity (52).

Of the 30 high-burden countries listed by the World Health Organization, 22 are elephant range countries, as well as supporting other iconic and endangered wildlife populations, including African wild dogs, mountain gorillas, Malaysian tigers, and black and Sumatran rhinoceros (1, 53). Due to the logistical difficulties in locating carcasses of wild animals for disease surveillance, most of the available information on risk of inter-species TB transmission originates from studies with captive wildlife (16). In Nepal, more than 10 working elephants have died of TB with the isolate identified as belonging to a Mtb lineage found among human patients in that country (30). Studies of TB in elephants in India, Thailand, Sri Lanka, Malaysia, Nepal and Laos have also confirmed transmission between humans and elephants (11, 30, 33, 54–56). Mtb has also been found in other wildlife in these countries, including free-living hanuman langurs in India and free-ranging and captive macaques in four Asian countries (10, 57).

Data on Mtb cases in African wildlife is limited compared to that in Asian species. *Mycobacterium tuberculosis* was diagnosed in eight species of wildlife at the National Zoological Gardens in South Africa (7). Since that report, there have been two deaths of African elephants at this zoo attributed to Mtb (unpubl. data). In addition, an increase in cases of Mtb documented in captive wildlife facilities in South Africa (including antelope, primates and warthogs) between 2002 and 2011, suggests greater spill-over in parallel with the growing human epidemic (58).

Disease surveillance is essential to determine the presence and extent of Mtb infection in wildlife, however this is limited by the logistical challenges associated with acquiring fresh samples from carcasses. This can be overcome by ante-mortem testing, although there is a paucity of diagnostic tests available for TB detection in wildlife. Serological assays and trunk wash sampling for mycobacterial cultures have been used to identify infected elephants (13, 15, 48). As demonstrated in this case, serological tests using MTBC-specific antigens for antibody detection could provide a presumptive diagnosis of TB in elephants, with reportedly higher sensitivity as compared to trunk wash culture (18, 22, 29, 55). Surveys of TB in elephants using serological tests have reported high prevalence in at-risk populations. For example, in the Malaysian peninsula, elephants had a seroprevalence of 20.4% and their handlers, tested with an interferon-gamma release assay, had a prevalence of 24.8% (33). Similarly, 13% of Nepal's and 15% of India's captive elephant populations were seropositive in recent surveys (35, 51, 54). High prevalence in these populations highlights the significant risk that humans present to captive and, potentially, free-ranging wildlife at interfaces.

Advances in molecular epidemiological and other diagnostic techniques have facilitated understanding of inter-species transmission (29, 58). Whole genome sequencing provides a tool for determining potential sources, number and timing of introduction of pathogens to a population. Although such tools have been commonly used in disease outbreaks in humans (59), they have been under-utilized in conservation efforts for evaluation of risks at interfaces. In addition to Mtb, other human pathogens such as methicillin-resistant *Staphylococcus aureus*, influenza A virus, and protozoal organisms such as *Giardia* and *Cryptosporidium*, present under-recognized biological threats to wildlife and domestic animal species (4). Since many outbreaks occur in developing countries where there is a lack of diagnostic capacity, strengthening collaboration between human and veterinary diagnostic centers would provide expertise and maximize use of limited resources (3). As the human-animal interface increases in complexity and sheer size globally, a greater focus on building multi-disciplinary teams with expertise in veterinary medicine, conservation, animal management, ecology, epidemiology, public health, and infectious diseases are needed to address the threat that human pathogens present to animals, especially threatened and endangered species. Without a One Health approach to managing diseases such as TB, it is unlikely that public health goals will be achieved, since there is a possibility of spill-over and spill-back between species, and this may result in devastating consequences for ecosystems such as loss of biodiversity and impact on environmental health (52, 60).

## Author Contributions

MM, PB, GH, and LvS conducted the post-mortem examination. EM performed histopathological examination. MM, AS-G, and KL performed serological tests. ER, RW, and AD performed mycobacterial cultures and speciation. SR-A and AD performed whole genome sequencing and analyzed whole genome sequence data. MM, PB, ER, AD, PvH, SP, RW, WW, and KL had key input into project design. All authors assisted with writing and editing the manuscript.

### Conflict of Interest Statement

KL and AS-G are employed by Chembio Diagnostic Systems, Inc. The remaining authors declare that the research was conducted in the absence of any commercial or financial relationships that could be construed as a potential conflict of interest.

## References

[B1] World Health Organization Global Tuberculosis Report 2017. Available online at: http://www.who.int/tb/publications/global_report/en/ (Accessed May 12, 2018).

[B2] World Health Organization Country Health Statistics Website. Available online at: http://www.who.int/tb/data/ (Accessed May 12, 2018).

[B3] Halliday JE Allan KJ Ekwem D Cleaveland S Kazwala RR Crump JA. Endemic zoonoses in the tropics: a public health problem hiding in plain sight. Vet Rec. (2015) 176:220–5. 10.1136/vr.h798 25722334 PMC4350138

[B4] Messenger AM Barnes AN Gray GC. Reverse zoonotic disease transmission (zooanthroponosis): a systematic review of seldom-documented human biological threats to animals. PLoS ONE (2014) 9:e89055. 10.1371/journal.pone.0089055 24586500 PMC3938448

[B5] Ameni G Vordermeier M Firdessa R Aseffa A Hewinson G Gordon SV. *Mycobacterium tuberculosis* in infection in grazing cattle in central Ethiopia. Vet J. (2010) 188:359–61. 10.1016/j.tvjl.2010.05.005 20965132 PMC3103825

[B6] Erwin PC Bemis DA Mawby DI McCombs SB Sheeler LL Himelright IM. *Mycobacterium tuberculosis* transmission from human to canine. Emerg Infect Dis. (2004) 10:2258–60. 10.3201/eid1012.040094 15672533 PMC3323378

[B7] Michel AL Venter L Espie IW Coetzee ML. *Mycobacterium tuberculosis* infections in eight species at the National Zoological Gardens of South Africa, 1991-2001. J Zoo Wildl Med. (2003) 34:364–70. 10.1638/02-063 15077712

[B8] Miller M Lyashchenko K Mycobacterial Infections in Other Zoo Animals Ch. 15. In Mukundan H Chambers MA Waters WR Larsen MH, editors. Tuberculosis, Leprosy and Mycobacterial Diseases of Man and Animals – The Many Hosts of Mycobacteria. Boston, MA: CABI (2015). p. 277–95.

[B9] Schmidt V Schneider S Schlomer J Krautwald-Junghanns ME Ricter E. Transmission of tuberculosis between men and pet birds: a case report. Avian Pathol. (2008) 37:589–92. 10.1080/03079450802428901 18821184

[B10] Parmar SM Jani RG Kapadiya FM Sutariya DR Status of tuberculosis in the free living hanuman langur (*Presbytis entellus*) of Gujarat state. Indian Vet J. (2013) 90:74–5.

[B11] Perera BVP Salgadu MA Gunawardena GSP de S Smith NH Jinadasa HRN First confirmed case of fatal tuberculosis in a wild Sri Lankan elephant. Gajah (2014) 41:28–31.

[B12] Zachariah A Pandiyan J Madhavilatha GK Mundayoor S Chandramohan B Sajesh PK. *Mycobacterium tuberculosis* in wild Asian elephants, southern India. Emerg Infect Dis. (2017) 23:504–6. 10.3201/eid2303.161741 28221104 PMC5382741

[B13] Charlesworth KE Vogelnest L Stephens N Marks GB. Diagnosis, investigation and management of tuberculosis at an Australian zoo. NSW Pub Health Bull. (2013) 24:49. 10.1071/NB13003 23849031

[B14] Ghielmetti G Coscolla M Ruetten M Friedel U Loiseau C Feldmann J. Tuberculosis in Swiss captive Asian elephants: microevolution of *Mycobacterium tuberculosis* characterized by multilocus variable-number tandem-repeat analysis and whole-genome sequencing. Sci Rep. (2017) 7:14647. 10.1038/s41598-017-15278-9 29116204 PMC5676744

[B15] Murphree R Warkentin JV Dunn JF Schaffner W Jones T. Elephant-to-human transmission of tuberculosis, 2009. Emerg Infect Dis. (2011) 17:366–71. 10.3201/eid1703.101668 21392425 PMC3166032

[B16] Oh P Granich R Scott J Sun B Joseph M Stringfield C. Human exposure following *Mycobacterium tuberculosis* infection of multiple animal species in a metropolitan zoo. Emerg Infect Dis. (2002) 8:1290–3. 10.3201/eid0811.020302 12453358 PMC2738539

[B17] Lyashchenko KP Grandison A Keskinen K Sikar-Gang A Lambotte P Esfandiara J. Identification of novel antigens recognized by serum antibodies in bovine tuberculosis. Clin Vaccine Immunol. (2017) 24: e00259–17. 10.1128/CVI.00259-17 28978510 PMC5717178

[B18] Greenwald R Lyashchenko O Esfandiari J Miller M Mikota S Olsen JH. Highly accurate antibody assays for early and rapid detection of tuberculosis in African and Asian elephants. Clin Vaccine Immunol. (2006) 16:605–12. 10.1128/CVI.00038-09 19261770 PMC2681580

[B19] Goosen WJ Miller MA Chegou NN Cooper D Warren RM van Helden PD. Agreement between assays of cell-mediated immunity utilizing *Mycobacterium bovis*-specific antigens for the diagnosis of tuberculosis in African buffaloes (*Syncerus caffer*). Vet Immunol Immunopath. (2014) 160:133–8. 10.1016/j.vetimm.2014.03.015 24794136

[B20] Warren RM gey van Pittius NC Barnard M Hesseling A Engelke E de Kock M. Differentiation of *Mycobacterium tuberculosis* complex by PCR amplification of genomic regions of difference. Int J Tuberc Lung Dis. (2006) 10:818–22. 16850559

[B21] Warren R de Kock M Engelke E Myburgh R van Pittius NG Victor T Safe *Mycobacterium tuberculosis* DNA extraction method that does not compromise integrity. J Clin Micro. (2006) 44:254–6. 10.1128/JCM.44.1.254-256.2006 PMC1351970 16390984

[B22] Lyashchenko KP Greenwald R Esfandiari J Olsen JH Ball R Dumonceaux G. Tuberculosis in elephants: antibody responses to defined antigens of *Mycobacterium tuberculosis*, potential for early diagnosis, and monitoring treatment. Clin Vaccine Immunol. (2006) 13:722–32. 10.1128/CVI.00133-06 16829608 PMC1489565

[B23] Kamerbeek J Schouls LEO Kolk A Van Agterveld M Van Soolingen D Kuijper S. Simultaneous detection and strain differentiation of *Mycobacterium tuberculosis* for diagnosis and epidemiology. J Clin Micro. (1997) 35:907–14. 9157152 10.1128/jcm.35.4.907-914.1997 PMC229700

[B24] Victor TC de Haas PE Jordaan AM van der Spuy GD Richardson M van Soolingen D. Molecular characteristics and global spread of *Mycobacterium tuberculosis* with a Western Cape F11 genotype. J Clin Microbiol. (2004) 42:769–72. 10.1128/JCM.42.2.769-772.2004 14766851 PMC344472

[B25] Obanda V Poghon J Yongo M Mulei I Ngotho M Waititu K. First reported case of fatal tuberculosis in a wild African elephant with past human-wildlife contact. Epidem Infect. (2013) 141:1476–80. 10.1017/S0950268813000022 23340041 PMC9155287

[B26] Chandranaik BM Shivashankar BP Umashankar KS Nandini P Giridhar P Byregowda SM. *Mycobacterium tuberculosis* in free-roaming wild Asian elephant. Emerg Infect Dis. (2017) 23:555–7. 10.3201/eid2303.161439 28221114 PMC5382756

[B27] Hlokwe TM van Helden P Michel AL. Evidence of increasing intra and inter-species transmission of *Mycobacterium bovis* in South Africa: are we losing the battle? Prev Vet Med. (2014) 115:10–7. 10.1016/j.prevetmed.2014.03.011 24703246

[B28] Michel AL Bengis RG Keet DF Hofmeyr M de Klerk L-M Cross PC. Wildlife tuberculosis in South African conservation areas: implications and challenges. Vet Micro. (2006) 112:91–100. 10.1016/j.vetmic.2005.11.035 16343819

[B29] Lewerin SS Olsson SL Eld K Roken B Ghebremichael S Koivula T. Outbreak of *Mycobacterium tuberculosis* infection among captive Asian elephants in a Swedish zoo. Vet Rec. (2005) 156:171–5. 10.1136/vr.156.6.171 15736698

[B30] Paudel S Mikota SK Nakajima C Gairhe KP Maharjan B Thapa J. Molecular characterization of *Mycobacterium tuberculosis* isolates from elephants of Nepal. Tuberculosis (2014) 94:287–92. 10.1016/j.tube.2013.12.008 24566285

[B31] Simpson G Zimmerman R Shashkina E Chen L Richard M Bradford CM. *Mycobacterium tuberculosis* infection among Asian elephants in captivity. Emerg Infect Dis. (2017) 23:513–6. 10.3201/eid2303.160726 28221115 PMC5382730

[B32] Ocepek M Pate M Zolnir-Dove M Poljak M. Transmission of *Mycobacterium tuberculosis* from human to cattle. J Clin Micro. (2013) 43:3555–7. 10.1128/JCM.43.7.3555-3557.2005 16000505 PMC1169140

[B33] Ong BL Ngeow YF Razak MA Yakubu Y Zakaria Z Mutalib AR. Tuberculosis in captive Asian elephants (*Elephas maximus*) in Peninsular Malaysia. Epidem Infect. (2013) 141:1481–7. 10.1017/S0950268813000265 23414617 PMC9151606

[B34] Romero B Rodriguez S Bezos J Diaz R Copano MF Merediz I Humans as source of *Mycobacterium tuberculosis* infection in cattle, Spain. Emerg Inf Dis. (2011) 12:2393–5. 10.3201/eid1712.101476 PMC3311187 22172249

[B35] Pavlik I Ayele WY Parmova I Melicharek I Hanzilkova M Svenjnochova M *Mycobacterium tuberculosis* in animal and human populations in six Central European countries during 1990-1999. Vet Med Czech (2003) 43:83–9.

[B36] Feldman M Isaza R Prins C Hernandez J. Point prevalence and incidence of *Mycobacterium tuberculosis* complex in captive elephants in the United States. Vet Q (2013) 33:28–32. 10.1080/01652176.2013.772690 23477422 PMC3631271

[B37] Abraham D Pillai VKP Cross-species transmission of *Mycobacterium tuberculosis* in mahouts and captive elephants: implications to health policy. Intern J Infect Dis. (2016) 45S:463 10.1016/j.ijid.2016.02.981

[B38] Stephens N Vogelnest L Lowbridge C Christensen A Marks GB Sintchenko V. Transmission of *Mycobacterium tuberculosis* from an Asian elephant (*Elephas maximus*) to a chimpanzee (*Pan troglodytes*) and humans in an Australian zoo. Epidem Inf. (2013) 141:1488–97. 10.1017/S095026881300068X 23537562 PMC9151603

[B39] Barasona JA Vicente J Diez-Delgado I Aznar J Gortázar C Torres MJ. Environmental presence of *Mycobacterium tuberculosis* complex in aggregation points at the wildlife/livestock interface. Transb Emerg Dis. (2016) 64:1148–58. 10.1111/tbed.12480 26865411

[B40] Garnett BT Delahay RJ Roper TJ. Use of cattle farm resources by badgers (*Meles meles*) and risk of bovine tuberculosis (*Mycobacterium bovis*) transmission to cattle. Proc Royal Soc London B Biol Sci. (2002) 269:1487–91. 10.1098/rspb.2002.2072 12137579 PMC1691052

[B41] Ribeiro-Lima J Carstensen M Cornicelli L Forester JD Wells SJ Patterns of cattle farm visitation by white-tailed deer in relation to risk of disease transmission in a previously infected areas with bovine tuberculosis in Minnesota, USA. Transb Emerg Dis. (2017) 64:1519–29. 10.1111/tbed.12544 27393719

[B42] Romero B Aranaz A Sandoval Á Álvarez J De Juan L Bezos J. Persistence and molecular evolution of *Mycobacterium bovis* population from cattle and wildlife in Doñana National Park revealed by genotype variation. Vet Micro. (2008) 132:87–95. 10.1016/j.vetmic.2008.04.032 18539410

[B43] Palmer MV Waters WR Whipple DL. Investigation of the transmission of *Mycobacterium bovis* from deer to cattle through indirect contact. Am J Vet Res. (2004) 65:1483–9. 10.2460/ajvr.2004.65.1483 15566085

[B44] Orumwense PO Torvinen E Heinonen-Tanski H. The survival of mycobacteria in pure human urine. Water Sci Technol. (2013) 67:1773–7. 10.2166/wst.2013.052 23579832

[B45] Laktis K Popluhar L Arvay S Current epizootiological situation of tuberculosis in Slovakia. Veterinarstvi (1970) 20:341–5.

[B46] Golub JE Cronin WA Obasanjo OO Coggin W Moore K Pope DS. Transmission of *Mycobacterium tuberculosis* through casual contact with an infectious case. Arch Int Med. (2001) 161:2254–8. 10.1001/archinte.161.18.2254 11575983

[B47] Golub JE Bur S Cronin WA Gange S Baruch N Comstock GW. Delayed tuberculosis diagnosis and tuberculosis transmission. Interntl J Tuberc Lung Dis. (2006) 10:24–30. 16466033

[B48] Johnson KR Braden CR Cairns KL Field KW Colombel AC Yang Z. Transmission of *Mycobacterium tuberculosis* from medical waste. JAMA (2000) 284:1683–8. 10.1001/jama.284.13.1683 11015799

[B49] Thapa J Nakajima C Gairhe KP Maharjan B Paudel S Shah Y Wildlife tuberculosis: an emerging threat for conservation in South Asia. Global exposition of wildlife management, Gbolagade Akeem Lameed. IntechOpen (2017). 10.5772/65798 [Epub ahead of print].

[B50] Mikota SK Maslow JN. Tuberculosis at the human-animal interface: an emerging disease of elephants. Tuberculosis (2011) 91:208–11. 10.1016/j.tube.2011.02.007 21397564

[B51] Mikota SK Gairhe K Giri K Hamilton K Miller M Paudel S Tuberculosis surveillance of elephants (*Elephas maximus*) in Nepal at the captive-wild interface. Eur J Wildl Res. (2015) 61:221–9. 10.1007/s10344-014-0890-4

[B52] Daszak P Cunningham AA Hyatt AD. Emerging infectious diseases of wildlife – threats to biodiversity and human health. Science (2000) 287:443–49. 10.1126/science.287.5452.443 10642539

[B53] International Union for Conservation of Nature Red List of Threatened Species. IUCN (2018). Available online at: http://www.iucnredlist.org/ (Accessed May 12, 2018).

[B54] Abraham D Cheeran JV Sukumar R Mikota SK Rao S Ganguly S Health Assessment of Captive Asian elephants in India With Special Reference to Tuberculosis. Project Elephant. Ministry of Environment and Forests. Government of India (2008).

[B55] Angkawanish T Wajjwalku W Sirimalaisuwan A. *Mycobacterium tuberculosis* infection of domesticated Asian elephants, Thailand. Emerg Infect Dis. (2010) 16:1949–51. 10.3201/eid1612.100862 21122228 PMC3294569

[B56] Lassausaie J Bret A Bouapao X Chanthavong V Castonguay-Vanier J Quet F Tuberculosis in Laos, who is at risk: the mahouts or their elephants? Epidemiol Infect. (2014) 143:922–31. 10.1017/S09502688.14002.180 25170549 PMC9507153

[B57] Wilbur AK Engel GA Rompis A Putra IA Lee BPH Aggimarangsee N From the mouths of monkeys: detection of *Mycobacterium tuberculosis* complex DNA form buccal swabs of synanthropic macaques. Am J Primat. (2012) 74:676–86. 10.1002/ajp.22022 PMC3368330 22644580

[B58] Michel AL Hlokwe TM Espie IW Zijll Langhout M Koeppel K Lane E. *Mycobacterium tuberculosis* at a human/wildlife interface in a high TB burden country. Transb Emerg Dis. (2013) 60:46–52. 10.1111/tbed.12099 24171848

[B59] Walker TM Ip CL Harrell RH Evans JT Kapatai G Dedicoat MJ. Whole-genome sequencing to delineate *Mycobacterium tuberculosis* outbreaks: a retrospective observational study. Lancet Inf Dis. (2013) 13:137–46. 10.1016/S1473-3099(12)70277-3 23158499 PMC3556524

[B60] Paudel S Tsubota T Tuberculosis in elephants: a zoonotic disease at the human-elephant interface. Jap J Zoo Wildl Med. (2016) 21:65–9. 10.5686/jjzwm.21.65

