# Supplementary material for: Fatal Tuberculosis in a Free-Ranging African Elephant and One Health Implications of Human Pathogens in Wildlife
Source: Front Vet Sci. 2019 Feb 6;6:18. doi: 10.3389/fvets.2019.00018 (PMC6373532; doi:10.3389/fvets.2019.00018)
Supplement: Supplementary file 1 [file Data_Sheet_1.docx]

**Fatal Tuberculosis in a Free-Ranging African Elephant and One Health Implications of Human Pathogens in Wildlife**

Michele A. Miller^1*#^, Peter Buss^2*#^, Eduard O. Roos^1^, Guy Hausler^1^, Anzaan Dippenaar^1^, Emily Mitchell^3,4^, Louis van Schalkwyk^5^, Suelee Robbe-Austerman^6^, W. Ray Waters^7^, Alina Sikar-Gang^8^, Konstantin P. Lyashchenko^8^, Sven D.C. Parsons^1^, Robin Warren^1^, Paul van Helden^1^

^1^DST-NRF Centre of Excellence for Biomedical Tuberculosis Research, South African Medical Research Council Centre for TB Research, Division of Molecular Biology and Human Genetics, Faculty of Medicine and Health Sciences, Stellenbosch University, Cape Town, South Africa.

^2^Veterinary Wildlife Services, South African National Parks, Kruger National Park, Skukuza, South Africa.

^3^Department of Research and Scientific Services, National Zoological Gardens of South Africa, Pretoria, South Africa.

^4^Faculty of Veterinary Science, University of Pretoria, Onderstepoort, South Africa. ^5^Department of Agriculture, Forestry and Fisheries, Skukuza State Veterinary Office, Skukuza, South Africa.

^6^National Veterinary Services Laboratories, Animal Plant Health Inspection Service, United States Department of Agriculture, Ames, IA, USA.

^7^National Animal Disease Center, Agricultural Research Service, United States Department of Agriculture, Ames, IA, USA.

^8^Chembio Diagnostic Systems, Inc., Medford, NY, USA.

#Co-first authors.

Correspondence:

Prof. Michele Miller

miller@sun.ac.za

Dr. Peter Buss

peter.buss@sanparks.org

Table S1. Sequences of *Mycobacterium* *spp*. isolates included in the phylogenetic analysis

| Isolate name | Original study reference | Accession number |
| --- | --- | --- |
| Elephant 17-008894 | This study | SRR6487127 |
| F11 SA4498 | This study | Pending |
| F11 SA5165 | This study | Pending |
| F11 Malawi | (1) | ERR036190 |
| T92 | (2) | SRX003589 |
| T17 | (2) | SRX005394 |
| 95_0545 | (2) | SRX007721 |
| K21 | (2) | SRX002001 |
| K67 | (2) | SRX002004 |
| K93 | (2) | SRX002005 |
| T67 | (2) | SRX007715 |
| T85 | (2) | SRX003590 |
| 00_1695 | (2) | SRX007716 |
| 98_1833 | (2) | SRX007718 |
| M4100A | (2) | SRX007719 |
| 91_0079 | (2) | SRX007720 |
| K49 | (2) | SRX002002 |
| GM_1503 | (2) | SRX012272 |
| 4783_04 | (2) | SRX007723 |
| K37 | (2) | SRX002003 |
| 544404 | (2) | SRX007725 |
| 11821_03 | (2) | SRX007724 |
| 4141_04 | (2) | SRX007726 |
| GM_0981 | (2) | SRX007722 |
| *M. canetti* | (2) | SRX002429 |
| *M. orygis* | SRA EBI Animal | ERR015582 |
| *M.bovis* (ravenel) | SRA EBI Animal | SRR022532 |
| 541504 | (3) | ERR031459 |
| N0092 | (3) | ERR031480 |
| N0115 | (3) | ERR031484 |
| N0091 | (3) | ERR031479 |
| 533604 | (3) | ERS153831 |
| 823602 | (3) | ERS153832 |
| Mt256 | (4) | ERR181435 |
| H37Rv | (2) | ERS153830 |
| 150(3) BovisKNP | (5) | ERR1815542 |
| 440(5) BovisKNP | (5) | ERR1815545 |
| 659(A) BovisKNP | (5) | ERR1815546 |
| 734(16) BovisKNP | (5) | ERR1815547 |
| 1067(2) | (5) | ERR1815539 |
| 1081(1) | (5) | ERR1815540 |
| 1457(4) | (5) | ERR1815541 |
| 1531(10) | (5) | ERR1815543 |
| 1474 | (5) | ERR1815538 |
| 3912 (B) | (5) | ERR1815544 |
| *M. bovis* NI-1 | (6) | ERR125598 |
| *M. bovis* NI-2 | (6) | ERR125605 |
| *M. bovis* NI-3 | (6) | ERR125608 |
| *M. bovis* NI-4 | (6) | ERR125612 |
| *M. bovis* NI-5 | (6) | ERR125618 |
| *M. bovis* NI-6 | (6) | ERR125621 |
| *M. bovis* NI-7 | (6) | ERR125625 |
| *M. bovis* NI-8 | (6) | ERR125626 |
| *M. bovis* NI-9 | (6) | ERR125627 |
| *M. bovis* NI-10 | (6) | ERR125628 |
| *M. pinnipedii G01222* | (7) | SRR1239336 |
| *M. caprae D028* | (8) | SRR650227 |

**Supplementary methods**

Whole genome sequence data analyses

*Pre-processing, alignment and variant calling*

The Illumina paired-end reads published in this study (BioProject ID: PRJNA430907, PRJEB30969) and 53 genomes published previously or available in public databases (Table S1) were analysed as previously described^5^. Briefly, sequencing reads were trimmed based on phred quality scores with Trimmomatic^9^. Reads were aligned to the *Mycobacterium tuberculosis* H37Rv (GenBank NC000962.3) reference genome to detect genomic variants. Three alignment algorithms were used, namely, Novoalign (Novocraft), Burrows-Wheeler Aligner (BWA)^10^, and SMALT^11^. An average of more than 65x depth of coverage was obtained for all isolates sequenced for this study. The Genome Analysis Tool Kit (GATK)^12^ was used to identify single nucleotide variants in all of the alignment files from the three mapping algorithms used. Variants identified by the GATK in all three alignments that correspond in position and base identity were annotated and further filtered to exclude variants in *pe/ppe* family genes, repeat regions, insertion sequences and phages, as described previously^3^. Variants supported by at least 80% of reads were included for phylogenetic analysis.

*Phylogenetic analysis*

Concatenated sequences containing 31 417 high-confidence variable sites (coding and non-coding single nucleotide variants) were used to create a maximum likelihood phylogeny with RaxML using 1000 bootstrap pseudo-replicates^13^. The General time reversal nucleotide substitution model was applied for phylogenetic inference.

**References**

1. Guerra-Assuncao JA, Houben RM, Crampin AC, Mzembe T, Mallard K, Coll F, et al. Recurrence due to relapse or reinfection with *Mycobacterium tuberculosis*: a whole-genome sequencing approach in a large, population-based cohort with a high HIV infection prevalence and active follow-up. J Infect Dis (2015) 211(7):1154-1163.

2. Comas I, Chakravartti J, Small PM, Galagan J, Niemann S, Kremer K, et al. Human T cell epitopes of *Mycobacterium tuberculosis* are evolutionarily hyperconserved. Nat Genet (2010) 42(6):498-503.

3. Coscolla M, Lewin A, Metzger S, Maetz-Rennsing K, Calvignac-Spencer S, Nitsche A, et al. Novel *Mycobacterium tuberculosis* complex isolate from a wild chimpanzee. Emerg Infect Dis (2013) 19(6):969-976.

4. Blouin Y, Hauck Y, Soler C, Fabre M, Vong R, Dehan C, et al. Significance of the identification in the Horn of Africa of an exceptionally deep branching *Mycobacterium tuberculosis* clade. PLoS One (2012) 7(12):e52841.

5. Dippenaar A, Parsons SDC, Miller MA, Hlokwe T, gey van Pittius NC, Adroub SA, et al. Progenitor strain introduction of *Mycobacterium bovis* at the wildlife-livestock interface can lead to clonal expansion of the disease in a single ecosystem. Infect Genet Evol (2017) 51:235-238.

6. Biek R, O'Hare A, Wright D, Mallon T, McCormick C, Orton RJ, et al. Whole genome sequencing reveals local transmission patterns of *Mycobacterium bovis* in sympatric cattle and badger populations. PLoS Pathog (2012) 8(11):e1003008.

7. Bos KI, Harkins KM, Herbig A, Coscolla M, Weber N, Comas I, et al. Pre-Columbian mycobacterial genomes reveal seals as a source of New World human tuberculosis. Nature (2014) 514(7523):494-497.

8. Domogalla J, Prodinger WM, Blum H, Krebs S, Gellert S, Muller M, et al. Region of difference 4 in alpine *Mycobacterium caprae* isolates indicates three variants. J Clin Microbiol (2013) 51(5):1381-1388.

9. Bolger AM, Lohse M, Usadel B. Trimmomatic: a flexible trimmer for Illumina sequence data. Bioinformatics (2014) 30(15):2114-2120.

10. Li H, Durbin R. Fast and accurate short read alignment with Burrows-Wheeler transform. Bioinformatics (2009) 25(14):1754-1760.

11. Ponstingl H, Ning Z, editors. SMALT - A New Mapper for DNA Sequencing Reads. Intelligent Systems for Molecular Biology 2010 meeting; 2010 02 August 20102010.

12. McKenna A, Hanna M, Banks E, Sivachenko A, Cibulskis K, Kernytsky A, et al. The genome analysis toolkit: a MapReduce framework for analyzing next-generation DNA sequencing data. Genome Res. (2010) 20(9):1297-1303.

13. Stamatakis A. Using RAxML to infer phylogenies. Curr Protoc Bioinformatics (2015) 51(6):1.
